# Supplementary material for: The molecular dissection of TRIM25’s RNA-binding mechanism provides key insights into its antiviral activity
Source: Nat Commun. 2024 Oct 1;15:8485. doi: 10.1038/s41467-024-52918-x (PMC11445558; doi:10.1038/s41467-024-52918-x)
Supplement: Supplementary file 1 — Supplementary Information [file 41467_2024_52918_MOESM1_ESM.pdf]

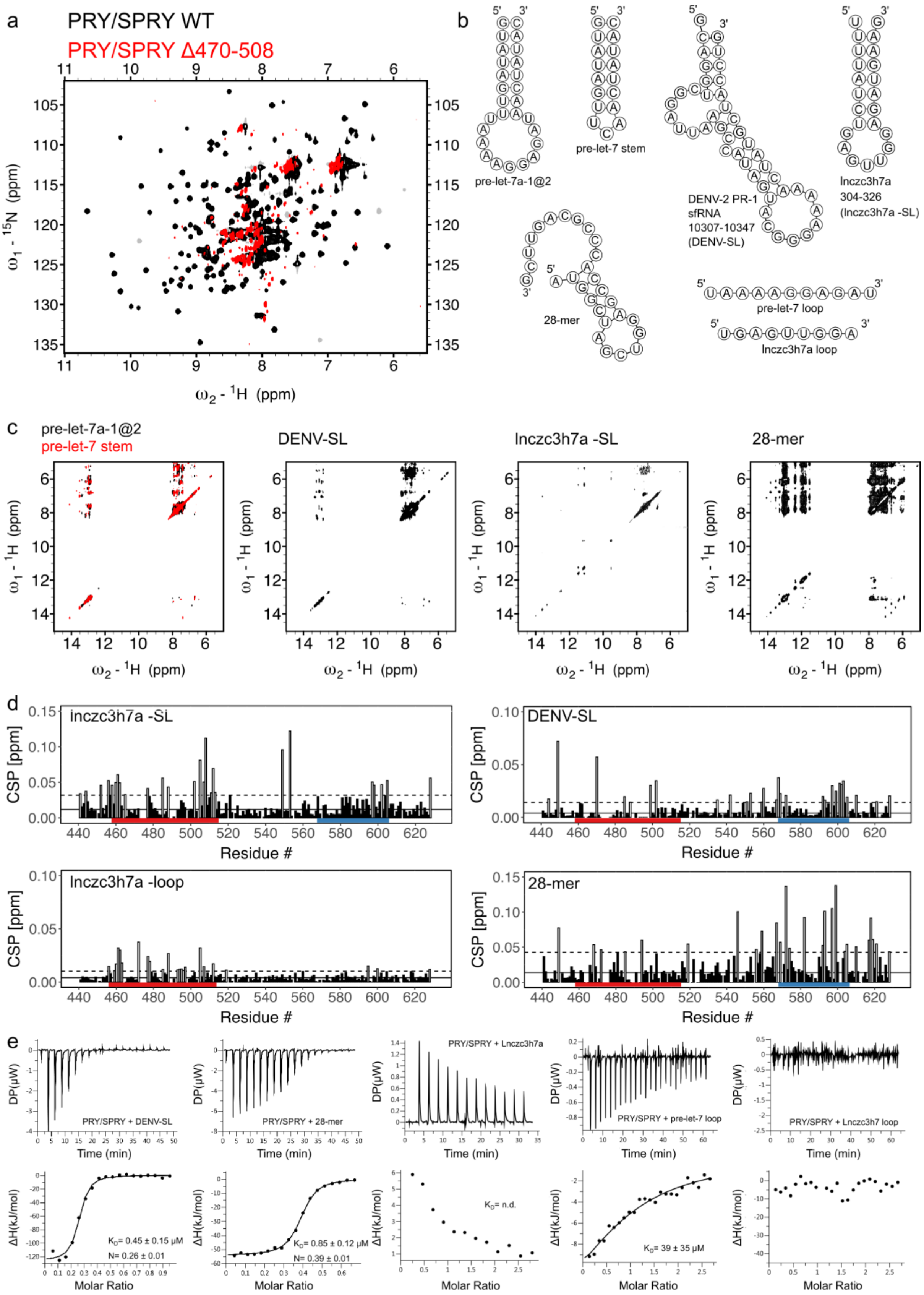
a
PRY/SPRY WT
PRY/SPRY Δ470-508
ω1 - 15N (ppm)
ω2 - 1H (ppm)
b
pre-let-7a-1@2
pre-let-7 stem
DENV-2 PR-1 sfRNA 10307-10347 (DENV-SL)
lnczc3h7a 304-326 (lnczc3h7a -SL)
28-mer
pre-let-7 loop
lnczc3h7a loop
c
pre-let-7a-1@2
pre-let-7 stem
DENV-SL
lnczc3h7a -SL
28-mer
ω1 - 1H (ppm)
ω2 - 1H (ppm)
d
lnczc3h7a -SL
DENV-SL
lnczc3h7a -loop
28-mer
CSP [ppm]
Residue #
e
PRY/SPRY + DENV-SL
PRY/SPRY + 28-mer
PRY/SPRY + Lnczc3h7a
PRY/SPRY + pre-let-7 loop
PRY/SPRY + Lnczc3h7 loop
DP(μW)
Time (min)
ΔH(kJ/mol)
Molar Ratio
KD= 0.45 ± 0.15 μM
N= 0.26 ± 0.01
KD= 0.85 ± 0.12 μM
N= 0.39 ± 0.01
KD= n.d.
KD= 39 ± 35 μM

f

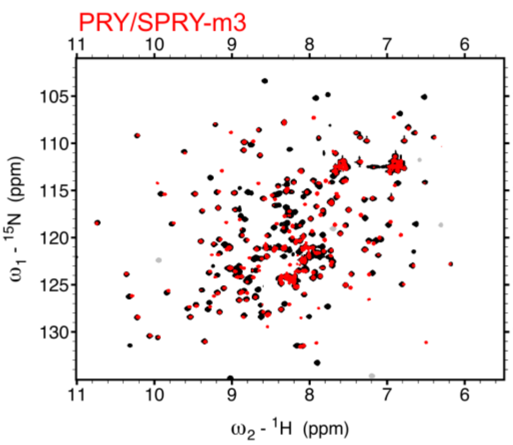

g

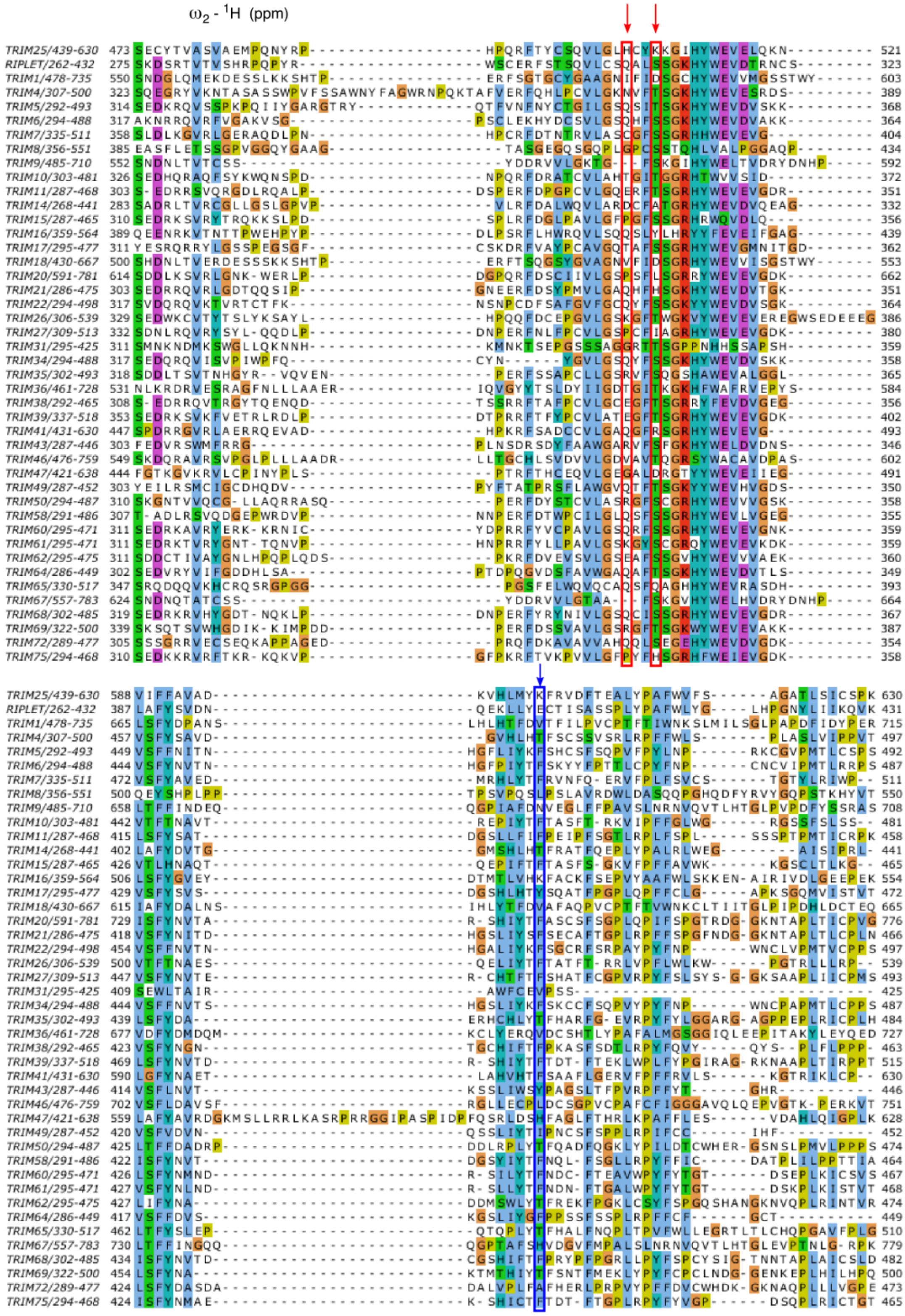

h

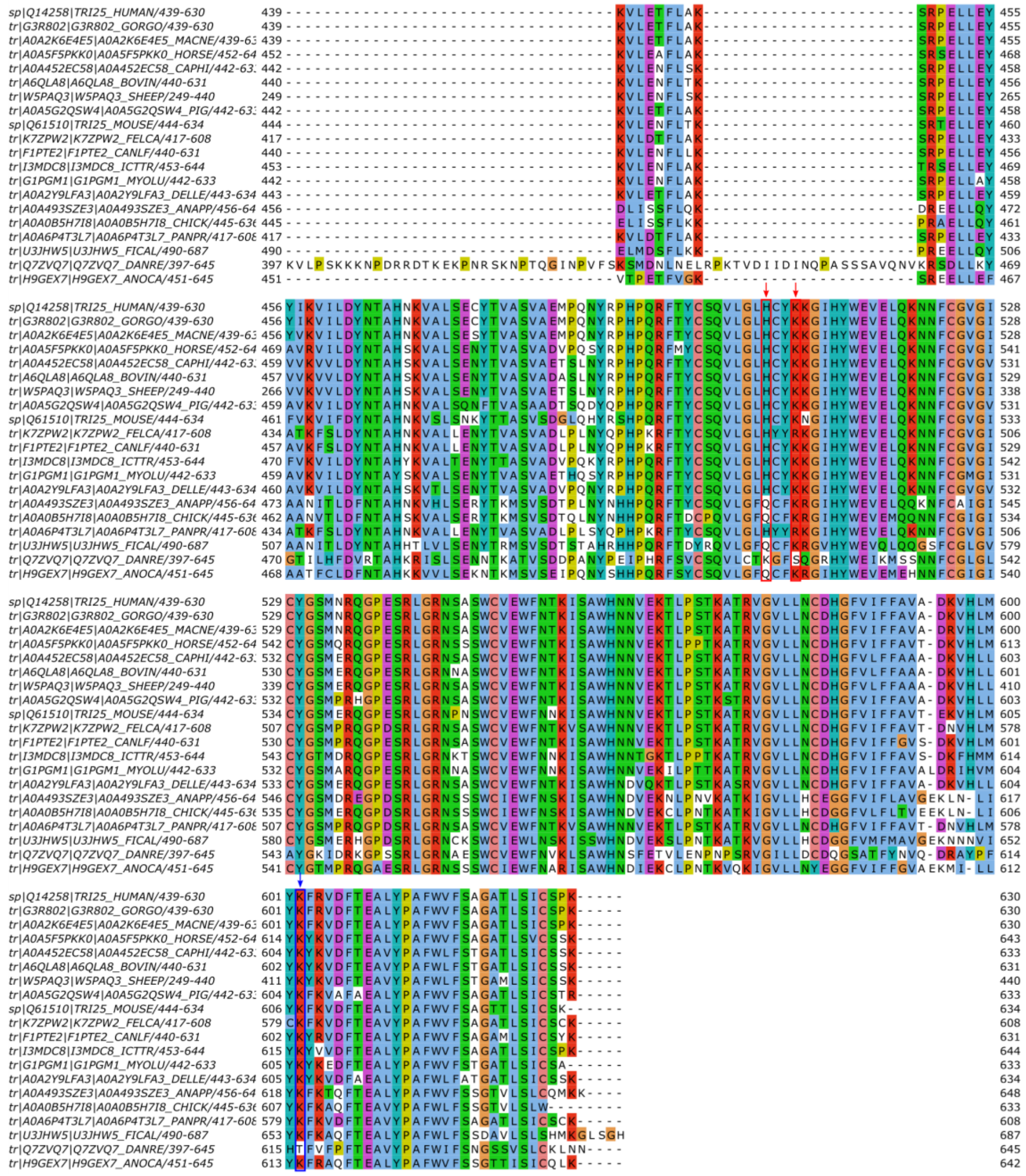

Supplementary Figure 1 (**a**) The ^{1}H/^{15}N-HSQC of the WT (black) and the previously described Δ470-508 deletion in the PRY/SPRY domain (ΔRBD by Choudhury et al. 2017[1], red) shows that the deletion construct is unfolded and prone to aggregation. **(b**) Predicted structures of the RNAs used in this study. All RNA structures were predicted using the RNAfold webserver.[2] **(c)** The observation of peaks in the imino region (10-15 ppm) in ^{1}H/^{1}H-2D-NOESYs confirm that stem-loops used in this study form double-stranded regions. Overlay of the NOESYs of pre-let-7a-1@2 (black) and the truncated pre-let-7 stem (red) show very similar imino-regions indicating that both constructs form double-stranded RNA with similar structures. (**d**) NMR titrations with different RNAs: The Dengue virus subgenomic stem-loop DENV-SL, the long non-coding RNA lnczc3h7a and a 28-mer duplex RNA show CSPs in binding site 1 and 2. The loop region of lnczc3h7a binds selectively to binding site 1 on the PRY/SPRY, similar to pre-let-7-loop, but with weaker affinity. The continuous line shows the average CSP and the dashed line indicates the average plus one standard deviation. Residues for which the CSPs are more than one standard deviation above the average are shown in open bars. Significantly affected regions are indicated in the protein sequence by red and blue horizontal bars. Source data are provided as a Source Data file. **(e)** Binding isotherms of TRIM25 PRY/SPRY titrated by DENV-SL, 28-mer, lnczc3h7a, pre-

let-7-loop and lnczc3h7a-loop. The value shown in the figure is the average of the different replicates and its standard deviation (n>2). All experimental setups and ITC measurements including replicates can be found in Supplementary Table 1. (**f**) The $^{1}H/^{15}N$-HSQC of the PRY/SPRY-m3 (red) described here is very similar to the wildtype (black) with only minor chemical shift perturbations of peaks corresponding to residues neighboring the mutation site. This indicates that structural changes are small and local. (**g**) Global alignment of the PRY/SPRY domain of all human TRIM proteins belonging to group IV[3] and Riplet (RNF135). The amino acid colour code used is the Clustal X colour scheme from Jalview, where hydrophobic residues are shown in blue, positive charges in red, negative charges in magenta, polar residues in green, cysteines in pink, glycines in orange, prolines in yellow and aromatic residues in cyan. The red arrows show the conservation of residues H505 and K508, belonging to binding site 1, and the blue arrow shows the conservation of K602, belonging to binding site 2. (**h**) Global alignment of the PRY/SPRY domain of TRIM25 proteins from 20 different species obtained from ENSEMBL (See material and methods for more information and Supplementary File Table 2 for details regarding the species). Conservation of sequence features of the PRY/SPRY domains within TRIM25 from different species. Residues responsible for RNA binding are shown in red or blue, depending on the binding site to which they belong. The height of the stack of letters at each position indicates the degree of conservation.

Supplementary Table 1. Summary of ITC-binding measurements.

Constructs	RNA	n	Conc. syringe (μM)	Conc. Cell (μM)	K_D (μM)	N	ΔH (kJ/mol)
PRY/SPRY	pre-let-7	4	1) 400; 2); 3) & 4) 200 (RNA)	1) 50; 2); 3) & 4) 35 (protein)	0.97 ± 0.20; 0.76 ± 0.21; 0.86 ± 0.34; 0.90 ± 0.25	0.36 ± 0.01; 0.33 ± 0.01; 0.33 ± 0.002; 0.31± 0.001	-95 ± 5
	DENV-SL	5	1) 250; 2); 3); 4) & 5) 200 (RNA)	1); 2); 3) & 4) 40<br>5) 20 (protein)	0.3 ± 0.1; 0.7 ± 0.4; 0.3 ± 0.2; 0.2 ± 0.1; 0. 6 ±0.1	0.27 ± 0.01; 0.29 ± 0.02; 0.22 ± 0.01; 0.24 ± 0.01; 0.28 ± 0.01	-67 ± 4
	28-mer	4	1) 800; 2); 3) & 4) 700(RNA)	1) 20; 2), 3) & 4) 200 (protein)	0.97 ± 0.15; 0.80 ± 0.11; 0.83 ± 0.07; 0.82 ± 0.13	0.37 ± 0.005; 0.40 ± 0.003; 0.38 ± 0.002; 0.41 ± 0.004	-55 ± 1
	Lnczc3h7a	3	1) ; 2) & 3) 750<br>4) 1100 (RNA)	50 (protein)	Weak binding K_D could not be determined		
	pre-let-7 loop	3	1) & 2) 800; 3) 700 (RNA)	1) & 2) 60<br>3) 50 (protein)	39 ± 30; 24 ± 22; 53 ± 49	-	-16 ± 9
	Lnczc3h7a loop	2	700 (RNA)	50 (protein)	No binding detected		
PRY/SPRY m3	pre-let-7	3	400 (RNA)	50 (protein)	No binding detected		
CC	pre-let-7	3	400 (RNA)	50 (protein)	3.2 ± 0.7; 3.0 ± 0.6; 3.4 ± 0.7	0.54 ± 0.02; 0.55 ± 0.01; 0.56 ± 0.02	-26 ± 1
	DENV-SL	3	1) 350; 2) 60; 3) 200 (RNA)	1) 50; 2) 10; 3) 30 (protein)	Not determined due to condensation		
	Lnczc3h7a	4	1) & 2) 300;<br>3) & 4) 600 (RNA)	1) & 2) 30;<br>3) 40 ; 4) 50 (protein)	3 ± 2; 1 ± 1; 1.6 ± 0.4; 1.5 ±0.4	-	-6.3 ± 0.8
	pre-let-7 loop	3	1) 800; 2) & 3) 1000 (RNA)	40 (protein)	49 ± 40; 38 ± 23; 84 ± 14	-	-14 ± 11
	Lnczc3h7a loop	3	1000 (RNA)	40 (protein)	59± 7; 61 ± 23; 63 ± 8	-	-40 ± 6
CC m2	pre-let-7	4	400 (RNA)	50 (protein)	5 ± 3; 6 ± 3; 2 ± 1; 4 ± 2	-	-3.6 ± 0.5
CC m4	pre-let-7	3	400 (RNA)	50 (protein)	No binding detected		
CC-PRY/SPRY	pre-let-7	3	100 (RNA)	10 (protein)	0.10 ± 0.06; 0.03 ± 0.03; 0.05 ± 0.05	1.14 ± 0.03; 1.09 ± 0.03; 1.06 ± 0.04	-27 ± 2
	DENV-SL	3	1) 60 2) & 3) 110 (CC-PRY/SPRY)	1) 6; 2) 10; 3) 10 (RNA)	KD_1 = 0.015 ± 0.004; 0.016 ± 0.002; 0.011 ± 0.003<br>KD_2 = 0.10 ± 0.04; 0.08 ± 0.02; 0.07 ± 0.02	N_1 = 0.78 ± 0.03; 0.87 ± 0.02; 0.79±0.02<br>N_2 = 0.08 ± 0.02; 0.12 ± 0.03; 0.010 ± 0.006	ΔH_1= -35 ± 3<br>ΔH_2= 299 ± 70
	Lnczc3h7a	3	1) & 2) 250; 3) 300 (RNA)	1) & 2) 25; 3) 20 (protein)	KD_1 = 0.25 ± 0.1; 0.03 ± 0.01; 0.02 ± 0.06<br>KD_2 = 2.9 ± 0.5; 1.2 ± 0.1; 0.3 ± 0.2	N_1 = 0.75 ± 0.11; 0.54 ± 0.01; 0.75 ± 0.1<br>N_2 = 0.42 ± 0.18; 0.55 ± 0.02; 0.47 ± 0.2	ΔH_1= 12 ± 7<br>ΔH_2= 58 ± 20
	pre-let-7 loop	2	1) 300; 2) 250 (RNA)	30 (protein)	3.5 ± 1.3; 3.9 ± 2.4	0.863 ± 0.05; 0.86 ± 0.09	-41 ± 7
	pre-let-7 stem	2	150 ((RNA)	20 (protein)	Weak binding K_D could not be determined		
	Lnczc3h7a loop	4	1); 2) & 3) 800;<br>4) 600 (RNA)	1) & 2) 50 ; 3) & 4) 25 (protein)	15 ± 7; 29 ± 3; 20 ± 5; 9 ± 4	1.8 ± 0.2; 1.8 ± 0.4.; 2.7 ± 0.1; 2.3 ± 0.2	-28 ± 6
	pre-let-7 modified	3	1) 100; 2) 300;<br>3) 269 (RNA)	1) 10; 2) 30; 3) 18 (protein)	KD_1 = 0.08 ± 0.03; 0.26 ± 0.02; 0.21 ± 0.05<br>KD_2 = 1.1 ± 0.02; 2.8 ± 0.1; 0.6 ± 0.04	N_1 = 0.80 ± 0.04; 1.04 ± 0.03; 0.60 ± 0.05<br>N_2 = 0.80 ± 0.03; 0.25 ± 0.05; 0.60 ± 0.05	ΔH_1= -19 ± 3<br>ΔH_2= -59 ± 7
	RNA_50_motif1 &2	2	1)100; 2) 150 (RNA)	1) 10; 2) 15 (protein)	KD_1 = 0.006 ± 0 .002; 0.0007 ± 0.01<br>KD_2 = 0.02±0.003; 0.2±0.02	N_1 = 0.36 ± 0.001; 0.54 ± 0.008<br>N_2 = 0.553 ± 0.001; 0.41 ±0.01	ΔH_1= -6.4 ± 4.6<br>ΔH_2= -42 ± 4
CC-PRY/SPRY 381-392 7KA	pre-let-7	3	1) 200; 2) & 3) 150 (RNA)	20 (protein)	KD_1 = 0.39 ± 0.22; 0.428 ± 0.055; 0.583 ± 0.072<br>KD_2 = 52 ± 16; 67 ± 12; 47 ± 4	N_1 = 0.61 ± 0.04; 0.765 ± 0.028; 0.624 ± 0.015<br>N_2 = 0.41 ± 0.39; 0.129 ± 0.218; 0.1 ± 0.1	ΔH_1= -24 ± 2<br>ΔH_2= -227 ±248
CC-PRY/SPRY m9	pre-let-7	3	200 (RNA)	20 (protein)	No binding detected		
	pre-let-7 modified	4	300 (RNA)	30 (protein)	No binding detected		

Supplementary Table 2. Animal species and Accession Numbers of the sequences used for the amino acid alignments shown in the Supporting Figure 1h. Data were obtained from the National Center for Biotechnology Information (NCBI).

**Common name**	**Animal**
Human	Homo sapiens
Gorilla	Gorilla gorilla
Macaque	Macaca nemestrina
Horse	Equus caballus
Goat	Capra hircus
Sheep	Ovis aries
Pig	Sus scrofa
Mouse	Mus Moluscos
Dog (domestic)	Canis lupus familiaris
Ground squirrel	Ictidomys tridecemlineatus
Little Brown bat	Myotis lucifugus
Beluga whale	Delphinapterus leucas
Duck	Anas platyrhynchos
Chicken	Gallus gallus
Leopard	Panthera pardus
Collared flycatcher	Ficedula albicollis
Zebrafish	Danio rerio
Green Anole	Anolis carolinensis

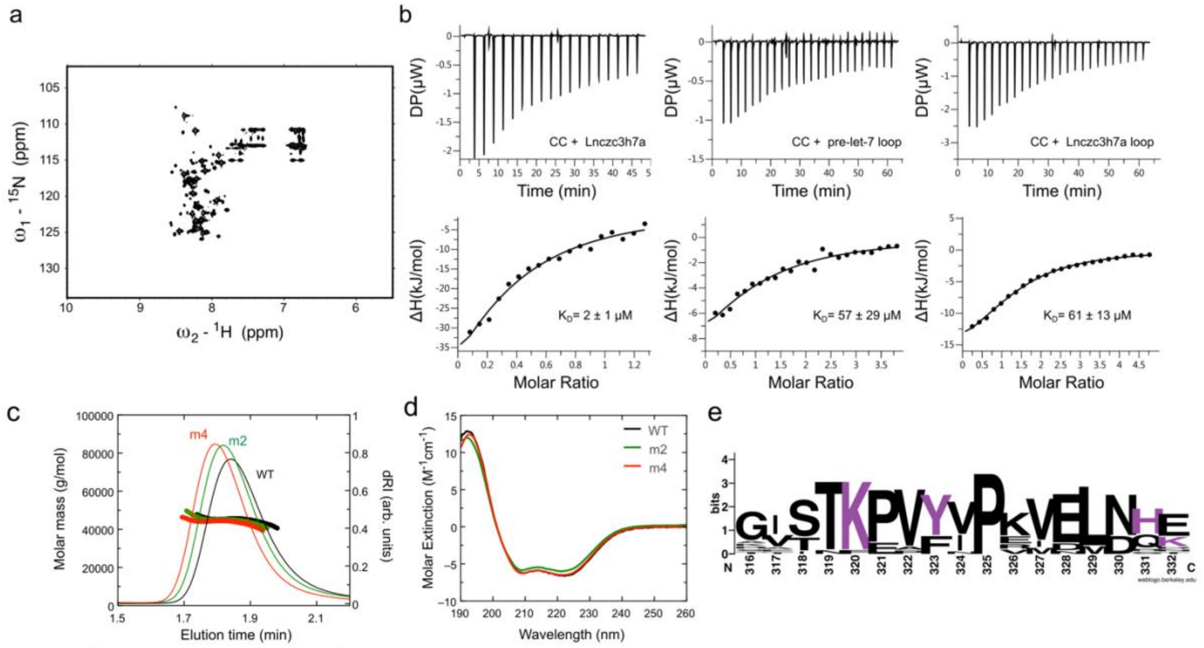

	theoretical MW (kDa)	experimental MW (kDa)
WT	43.945	44.8 ± 0.3
m2	43.865	44.5 ± 0.3
m4	43.717	43.6 ± 0.3

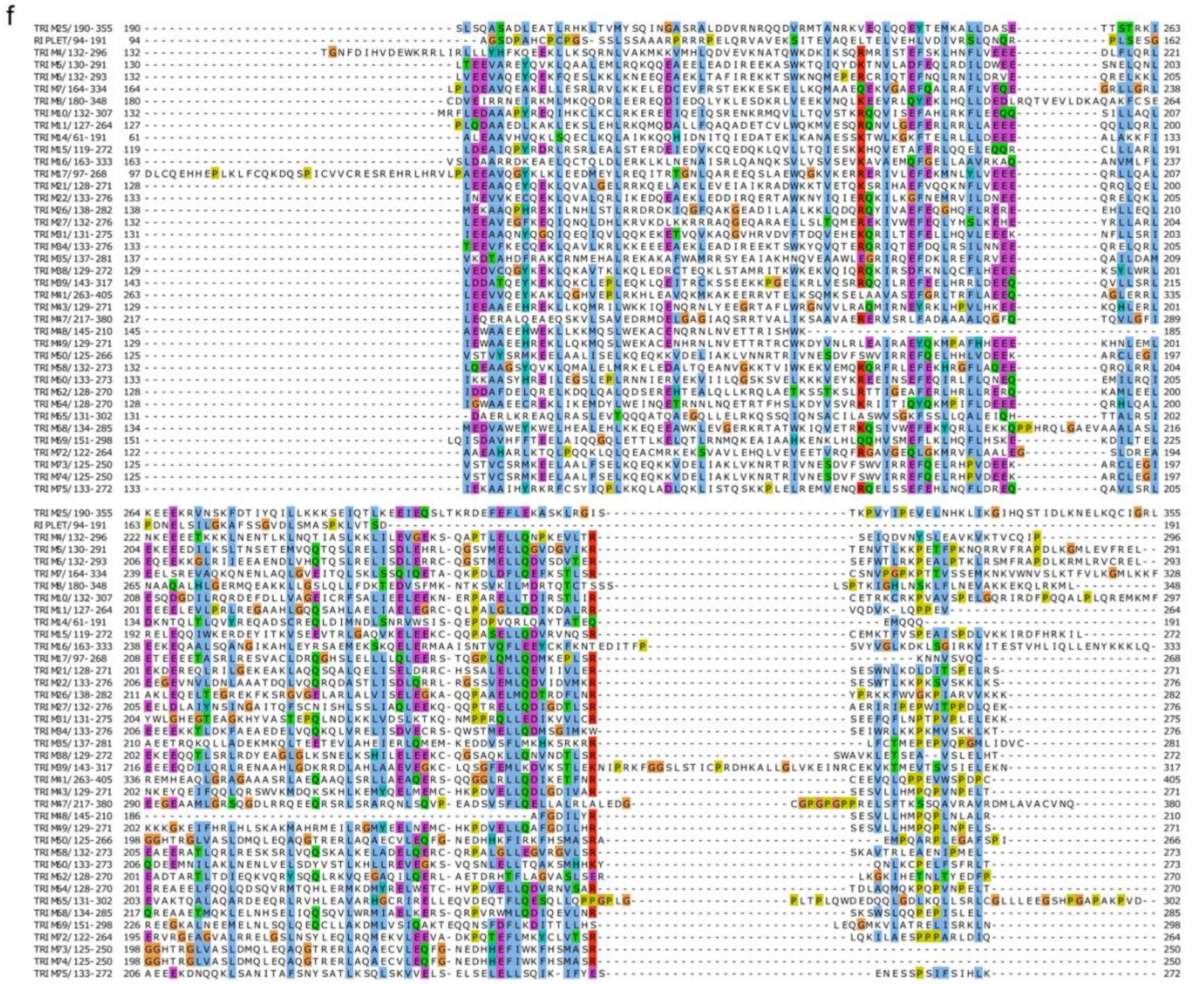

Supplementary Figure 2 (**a**) The ^{1}H,^{15}N-HSQC spectrum of the CC domain shows only peaks corresponding to residues of the flexible termini due to its elongated conformation and large size.

Therefore, NMR is not suitable for identifying RNA binding residues. (**b**) Binding isotherms of TRIM25 CC domain titrated with lnczc3h7a, pre-let-7 loop and lnczc3h7a-loop. The value shown in the figure is the average of all replicates (n >3). All experimental setups and ITC measurements including replicates are listed in Supplementary Table 1. (**c**) SEC-MALS analysis of the CC-WT domain (black) and its mutants (m2: green, m4: red). Protein concentrations were monitored using a refractive index detector (RI, lines). Mass weight determination (dots) indicates that the three samples are dimers in solution. (**d**) Circular dichroism spectroscopy curves for CC-WT and mutants showing no prominent changes in secondary structure content upon mutation. (**e**) Conservation of sequence features of residues within the CC domain peptide detected as RNA binding by CLIR-MS/MS experiments of TRIM25 from 18 different species (See material and methods for more information and Supplementary File Table 2 for details regarding the species). Residues responsible for RNA binding are shown in purple. The height of the letter stack at each position indicates the degree of conservation. (**f**) Global alignment of the CC domain of all human TRIM proteins belonging to group IV[3] and Riplet (RNF135). The amino acid colour code used is the Clustal X colour scheme from Jalview, where hydrophobic residues are shown in blue, positive charges in red, negative charges in magenta, polar residues in green, cysteines in pink, glycines in orange, prolines in yellow and aromatic residues in cyan.

Supplementary Table 3. Small-angle X-ray scattering statistics according to community guidelines in (Trewhella et al., 2017)[4].

	**TRIM25 CC-PRY/SPRY**	**TRIM25 CC-PRY/SPRY/pre-let-7**	**TRIM25 CC-PRY/SPRY/ lnczc3h7a**	**TRIM25 CC-PRY/SPRY/pre-let-7**
(a) Sample Details				
Organism	*Homo sapiens*			
Source	*E. coli* BL21 (DE3)	*E. coli* BL21 (DE3)/*in vitro* transcription		
Uniprot sequence ID	Q14258			
Description	TRIM25 189-630	TRIM25 189-630 in complex with the pre-let-7	TRIM25 189-630 in complex with the lnczc3h7a	TRIM25 189-630 in complex with the pre-let-7
Molecular mass M (Da)	49,952	58,195	57,358	58,195
Loading concentration (mg/ml)	0.37, 0.75, 1.5, 3 and 6	n.d.	n.d.	n.d.
Injection volume (µl)	30	100	30	500
Concentration (µM)	7.4, 15, 30, 60, 120	n.d.	n.d.	n.d.
Solvent composition and source	20 mM MES, pH 6.5, 75 mM NaCl and 0.5 mM TCEP			
(b) SAS data collection parameter				
Source and instrument	Hamburg PETRA-III P12 with Dectris Pilatus 6M			ESRF BM29 with Dectris Pilatus
Wavelength (Å)	1.22	1.24	1.24	0.99
Sample-detector distance (m)	3.0	3.0	3.0	2.869
q-measurement range (Å)	0.02267-7.405	0.0252-7.318	0.0297-7.267	0.0346-4.928
Radiation damage monitoring	frame-by-frame comparison			
Exposure time (s) & number	0.05x20	0.195x100	0.05x20	1s
Sample configuration	sample changer with flow through capillary measurement			SEC-SAXS, Superdex S200 10/300 Increase
Sample temperature (°C)	20	25	20	20
(c) Software employed for SAS data reduction, analysis and interpretation				
SAXS data processing	I(q) vs. q using Bsx cube, solvent subtraction and curve merging using PRIMUSqt from ATSAS (Franke et al., 2017)			I(q) vs. q using Bsx cube, solvent subtraction and curve merging Chromixs
Basic analyses: Guinier, PI, Vp	PRIMUSqt from ATSAS 2.7.1 (Franke et al., 2017)			
Atomic structure modelling	CRYSOL 2.8.2 from PRIMUSqt in ATSAS 2.8 (Svergun et al., 1995)			
Molecular graphics	-	-	-	-
(d) Structural parameters				
	Guinier analysis			
I(0) (raw)	51290 ± 160	34043 ± 42	0.033 ± 0.001	21.84 ± 0.22
R_g (Å)	68.3±0.5	56.3 ± 0.1	58.1 ± 1.8	57.0 ± 0.5
qR_g max	1.50	1.4	1.28	
Coefficient of correlation, R^2	0.82	0.80	0.77	0.77
	P(r) Analysis from AUTOGNOM			
I(0) (cm^{-1})	54320	35340	0.034	243
R_g (Å)	78.0	61.3	56.4	66.0
d_{max} (Å)	305.6	226.6	165.6	205.0
q range (Å^{-1})	0.202-3.06	0.148-2.50	0.145-1.38	0.394-3.31
χ^2 (total estimate from *GNOM*)	0.60	0.73	0.68	0.60
Porod volume (Å^{-3}) (ratio V_P/calculated M)	302540	182720	180000	215000
SASDB identifiers	SASDK78	SASDK88	SASDK8	SASDK98

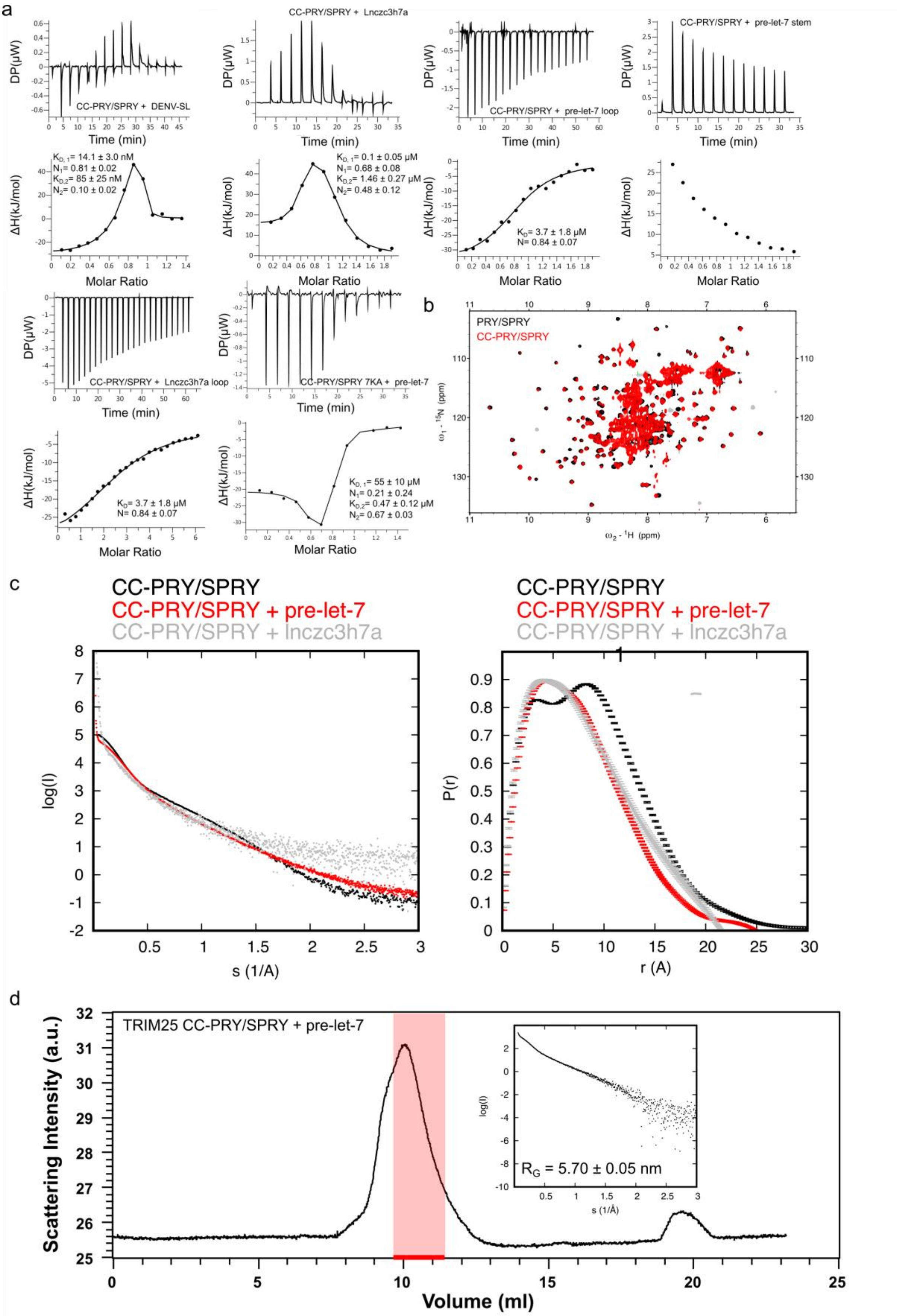
a
CC-PRY/SPRY + DENV-SL
CC-PRY/SPRY + Lnczc3h7a
CC-PRY/SPRY + pre-let-7 loop
CC-PRY/SPRY + pre-let-7 stem
DP(μW)
Time (min)
ΔH(kJ/mol)
Molar Ratio
K_D,1 = 14.1 ± 3.0 nM
N_1 = 0.81 ± 0.02
K_D,2 = 85 ± 25 nM
N_2 = 0.10 ± 0.02
K_D,1 = 0.1 ± 0.05 μM
N_1 = 0.68 ± 0.08
K_D,2 = 1.46 ± 0.27 μM
N_2 = 0.48 ± 0.12
K_D = 3.7 ± 1.8 μM
N = 0.84 ± 0.07
CC-PRY/SPRY + Lnczc3h7a loop
CC-PRY/SPRY 7KA + pre-let-7
K_D = 3.7 ± 1.8 μM
N = 0.84 ± 0.07
K_D,1 = 55 ± 10 μM
N_1 = 0.21 ± 0.24
K_D,2 = 0.47 ± 0.12 μM
N_2 = 0.67 ± 0.03
b
PRY/SPRY
CC-PRY/SPRY
ω1 - 15N (ppm)
ω2 - 1H (ppm)
c
CC-PRY/SPRY
CC-PRY/SPRY + pre-let-7
CC-PRY/SPRY + lnczc3h7a
log(I)
s (1/A)
P(r)
r (A)
d
TRIM25 CC-PRY/SPRY + pre-let-7
Scattering Intensity (a.u.)
Volume (ml)
log(I)
s (1/Å)
R_G = 5.70 ± 0.05 nm

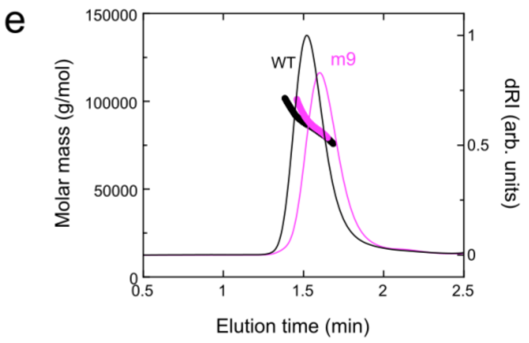

	theoretical MW (kDa)	experimental MW (kDa)
WT	50.106.	86.6 ± 0.3
m9	49.945	85.8 ± 0.3

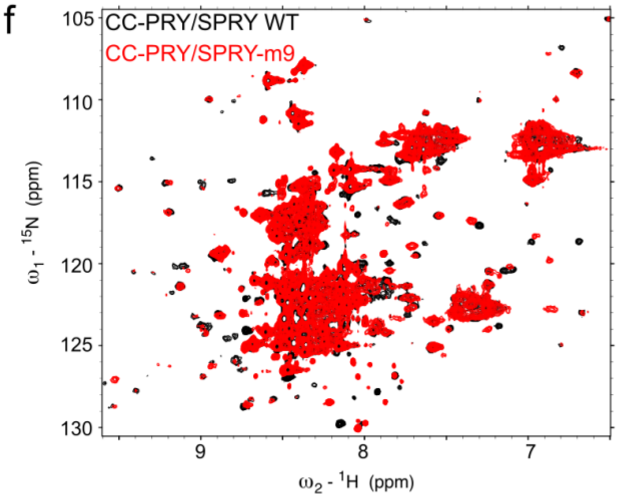

Supplementary Figure 3 (a) Binding isotherms of TRIM25 CC-PRY/SPRY domain titrated by DENV-SL, lnczc3h7a, pre-let-7 loop, pre-let-7 stem and lnczc3h7a loop and for TRIM25 CC-PRY/SPRY 7KA titrated by pre-let-7. The value shown in the figure is the average of all replicates and its standard deviation (n > 2). All experimental setups and the results of all ITC measurements including replicates can be found in Supplementary Table 1. **(b)** Overlay of the ^{1}H,^{15}N-HSQC spectra of TRIM25 PRY/SPRY (black) and CC-PRY/SPRY (red) shows excellent overlap for the dispersed peaks corresponding to folded regions. However, the signal intensity for these peaks is much weaker for CC-PRY/SPRY than for PRY/SPRY at the same concentration. Together with the absence of CSPs, this suggests that only the unbound state of PRY/SPRY is observed, while the state of PRY/SPRY bound to CC is not. **(c)** SAXS curves and pairwise distance distributions for free TRIM25 CC-PRY/SPRY (black) and its complex with pre-let-7 RNA (red) or lnczc3h7a-SL (gray). Binding of lnczc3h7a-SL leads to a similar reduction in radius of gyration and similar changes in the pairwise distribution function in SAXS as pre-let-7, indicating that stem-loop binding generally leads to the formation of a more compact complex, where the PRYSPRY is bound via RNA to the CC domain. **(d)** This is also confirmed by SEC-SAXS data showing that this reduction is not due to the presence of free RNA or aggregation artefacts. **(e)** SEC-MALS analysis of the CC-WT domain (black) and it mutant (m9, magenta). Protein concentrations were monitored using a refractive index detector (RI, lines). The weight determination (dots) shows that the two samples are a mixture of dimer and monomer in solution. **(f)** Superposition of the ^{1}H,^{15}N-HSQC spectra of TRIM25 CC-PRY/SPRY WT and m9 shows good overlap for most of the scattered peaks corresponding to folded regions. However, there is the presence of CSPs due to the mutations. **(g)** Overlay of the ^{1}H,^{15}N-HSQC spectra comparing CC-PRY/SPRY m9 in the absence (black) and presence (red) of pre-let-7 (3-fold excess). In contrast to TRIM25 CC-PRY/SPRY WT, there is no loss of signal from the peaks after the addition of RNA, confirming the ITC data that m9 does not bind RNA.

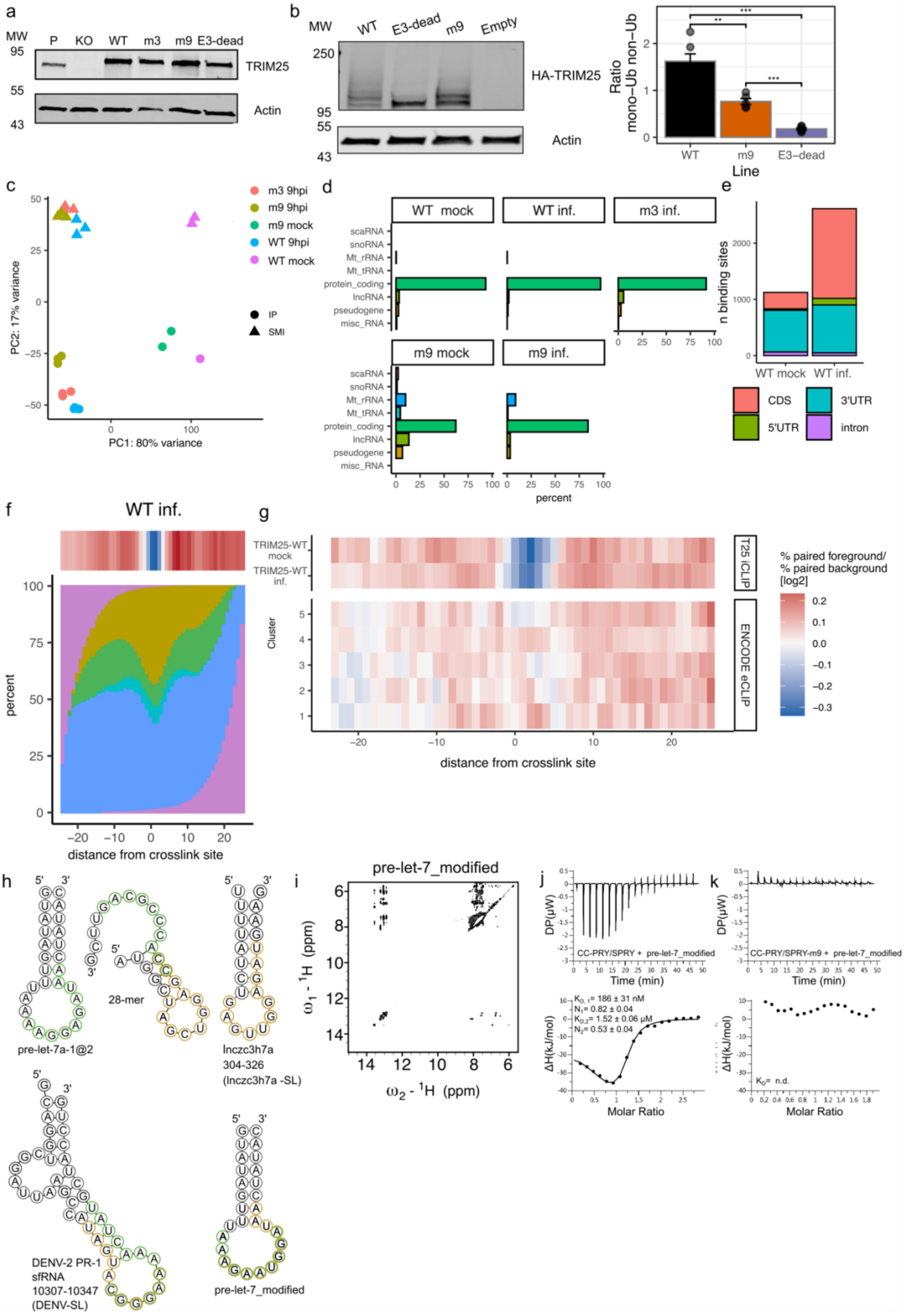
a
MW
P
KO
WT
m3
m9
E3-dead
95
55
43
TRIM25
Actin
b
MW
WT
E3-dead
m9
Empty
250
95
55
43
HA-TRIM25
Actin
Ratio mono−Ub non−Ub
Line
E3−dead
c
PC1: 80% variance
PC2: 17% variance
m3 9hpi
m9 9hpi
m9 mock
WT 9hpi
WT mock
IP
SMI
d
WT mock
WT inf.
m3 inf.
m9 mock
m9 inf.
scaRNA
snoRNA
Mt_rRNA
Mt_tRNA
protein_coding
lncRNA
pseudogene
misc_RNA
percent
e
n binding sites
CDS
3'UTR
5'UTR
intron
f
percent
distance from crosslink site
g
TRIM25-WT mock
TRIM25-WT inf.
Cluster
T25 iCLIP
ENCODE eCLIP
distance from crosslink site
% paired foreground/ % paired background [log2]
h
pre-let-7a-1@2
28-mer
lnczc3h7a 304-326 (lnczc3h7a -SL)
DENV-2 PR-1 sfRNA 10307-10347 (DENV-SL)
pre-let-7_modified
i
pre-let-7_modified
ω1 - 1H (ppm)
ω2 - 1H (ppm)
j
DP(μW)
CC-PRY/SPRY + pre-let-7_modified
Time (min)
KD,1= 186 ± 31 nM
N1= 0.82 ± 0.04
KD,2= 1.52 ± 0.06 μM
N2= 0.53 ± 0.04
ΔH(kJ/mol)
Molar Ratio
k
CC-PRY/SPRY-m9 + pre-let-7_modified
KD= n.d.

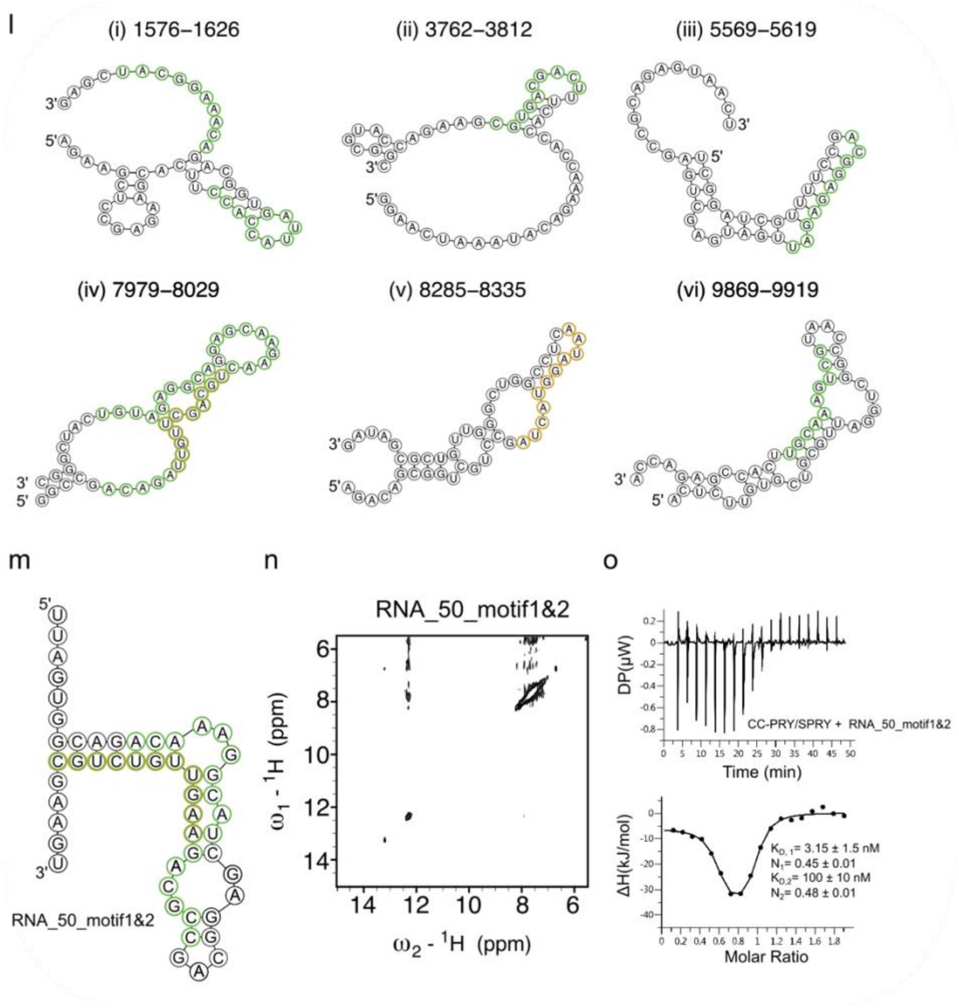

Supplementary Data Figure 4 (a) Western blot showing the parental cell line (P, HEK293), TRIM25 knockout (KO) and TRIM25 KO cells rescued with TRIM25-WT, TRIM25-m3, TRIM25-m9 and TRIM25-E3 ligase dead mutants. The presence or absence of protein bands in these lanes highlights the successful rescue by the different mutants and their effect on protein expression. Uncropped blots in Source Data. **(b)** *In vivo* ubiquitination assay of TRIM25 mutants. Quantification of the mono- and non-ubiquitination levels of six independent replicates (n=6; right panel) for TRIM25 WT (black); m9 (orange) and E3-dead (purple). Statistical differences are based on a two-tailed homoscedastic t-test (***$p < 0.001$; **$p < 0.01$; *$p < 0.05$). No adjustments were made for multiple comparisons. **c)** PCA of the different iCLIP2 sequencing data. **(d)** Bar plots showing the biotypes of RNAs identified by iCLIP2. **(e)** Absolute distribution of the binding sites across 5' UTRs, CDSs and 3' UTRs on cellular target RNAs for the different samples. **(f)** Percentage of paired and unpaired sequences across the binding site for the TRIM25-WT infected sample. **(g)** Clustering analysis of the percentage of paired and unpaired sequences across binding sites for 150 RBPs from the ENCODE eCLIP database yields 5 distinct clusters. Clusters 1 to 4 have paired RNA around the binding site; but differ in what happens further away from the binding site. The analysis for TRIM25-WT (top panel); both mock and infected; falls into cluster 5; which shows a unique pattern of unpaired RNA around the cross-linking site. **(h)** Mapping of the consensus motif identified for iCLIP2-identified cellular RNAs to the RNAs used in the biophysical study. **(i)** $^{1}H/^{1}H$-2D-NOESY spectrum for the RNA designed in which the two motifs were present (pre-let-7_modified) confirming the double-stranded regions. **(j)** Binding isotherms of TRIM25 CC-PRY/SPRY domain titrated by RNA where the two motifs were present (pre-let-7_modified). The value shown in the figure is the average of all replicates and its standard deviation. **(k)** Representative ITC binding isotherm for the TRIM25 CCPRY/SPRY-m9 (pre-let-7_modified complex (n=3). **(l)** Secondary structure with the sequence of the binding sites described in Figure 4h. The line surrounding the nucleotide indicates the presence of the identified motifs in the RNA structure (green for motif 1 and

orange for motif 2).**(m)** Secondary structure predicted for the RNA_50_motif1&2 with the line surrounding the nuclotides indicating the presence of the identified motifs. **(n)** $^{1}H/^{1}H$-2D-NOESY spectrum for the RNA designed in which the two motifs were present (RNA_50_motif1&2) confirming the double-stranded regions. **(o)** Binding isotherms of TRIM25 CC-PRY/SPRY domain titrated by RNA where the two motifs were present (RNA_50_motif1&2). The value shown in the figure is the average of all replicates. All experimental setups and the results of all ITC measurements including replicates can be found in Supplementary Table 1.

Supplementary Table 4. The number of cycles used for the iCLIP2 experiments for each replicate.

Sample	Threshold cycles (CT)	Number of cycles
WT mock rep1	14	11
WT mock rep2	15	12
WT mock rep3	14	11
WT 9hpi rep1	15	12
WT 9hpi rep2	16	13
WT 9hpi rep3	14	11
m3 9hpi rep1	14	11
m3 9hpi rep2	15	12
m3 9hpi rep3	15	12
m9 mock rep1	21	18
m9 mock rep3	15	12
m9 9hpi rep1	16	13
m9 9hpi rep2	16	13
m9 9hpi rep3	15	12
SMI WT mock rep1	12	9
SMI WT mock rep2	11	8
SMI WT mock rep3	13	10
SMI WT 9hpi rep1	12	9
SMI WT 9hpi rep2	15	12
SMI WT 9hpi rep3	19	16
SMI m3 9hpi rep1	12	9
SMI m3 9hpi rep2	14	11
SMI m3 9hpi rep3	15	12
SMI m9 9hpi rep1	13	10
SMI m9 9hpi rep2	14	11
SMI m9 9hpi rep3	13	10

Supplementary Table 5. Binding sites of TRIM25-WT on SINV-RNA.

Coordinates	Sequence (5'--> 3')
1497-1547	AAACUGCUGCAGGUCUCGGAGGAAUUAGUCAUGGAGGCCAAGGCUGCUUU
1576-1626	AGAAGCUCCGAGAAGCACUU**CCACCAUUAG**UGGCAG**ACAAAGGCAU**CGAG
2080-2130	UUGACGUGGA**CAAGAAGCGU**UGCGUUAAG**AAGGAAGAAG**CCUCAGGUCUG
3762-3812	GGAACUAAAUACAGAAACCACCACUU**UCAGCAGUGC**GAAGACCAUGCGGC
4187-4237	AGGCGAAGGAGUCUGCCGUGCCAUCUAUAAACGUUGGCCGACCAGUUUUA
4320-4370	CGGAAGCAC**CCAGAAGCAG**AAGC**CUUGAAAUUGCUACAAAA**CGCCUACCA
5236-5286	GCUUUAGCGGAUCGGACAACUCUAUUACUAGUAUGGACAGUUGGUCGUCA
5569-5619	UCGGAUCGUUUUCCG**ACGGAGAGAU**UGAUGAGCUGAGCCGCAGAGUAACU
6931-6981	UGA**UCUUGGAGGACCUGGGUGU**GGAUCAACCACUACUCGACUUGAUCGAG
7414-7464	UGCUAGAUGAAACAAAGGCGUGGUUUAGAGUAGGUAUAACAGGCACUUUA
7546-7596	GAGCAUUCCAAGCCAUCAGAGGGGAAAUAAAGCAUCUCUACGGUGGUCCU
7979-8029	GGCCG**ACAGAUUGUUCGACGUCAAGAACGAG**G**ACGGAGAUGU**CAUCGGGC
8285-8335	AGACAGCGGUCGUCCG**AUCAUGGAUAA**CUCCGGUCGGGUUGUCGCGAUAG
8393-8443	UAAAGGGAAGACAAUUAAGACGACCCCGGAAGGG**ACAGAAGAGU**GGUCCG
9869-9919	ACUCUUGUGCUGCGUUAGGUCGGCCAAU**GCUGAAACGU**UCACCGAGACCA
8405-8455	AAUUAAGACGACCCCGGAAGGG**ACAGAAGAGU**GGUCCGCAGCACCACUGG

The colour of the letter indicates the presence of the motifs (motif 1 in green; motif 2 in orange) identified by iCLIP2 for the cellular binding sites. Underlined nucleotides indicate the presence of both motifs.

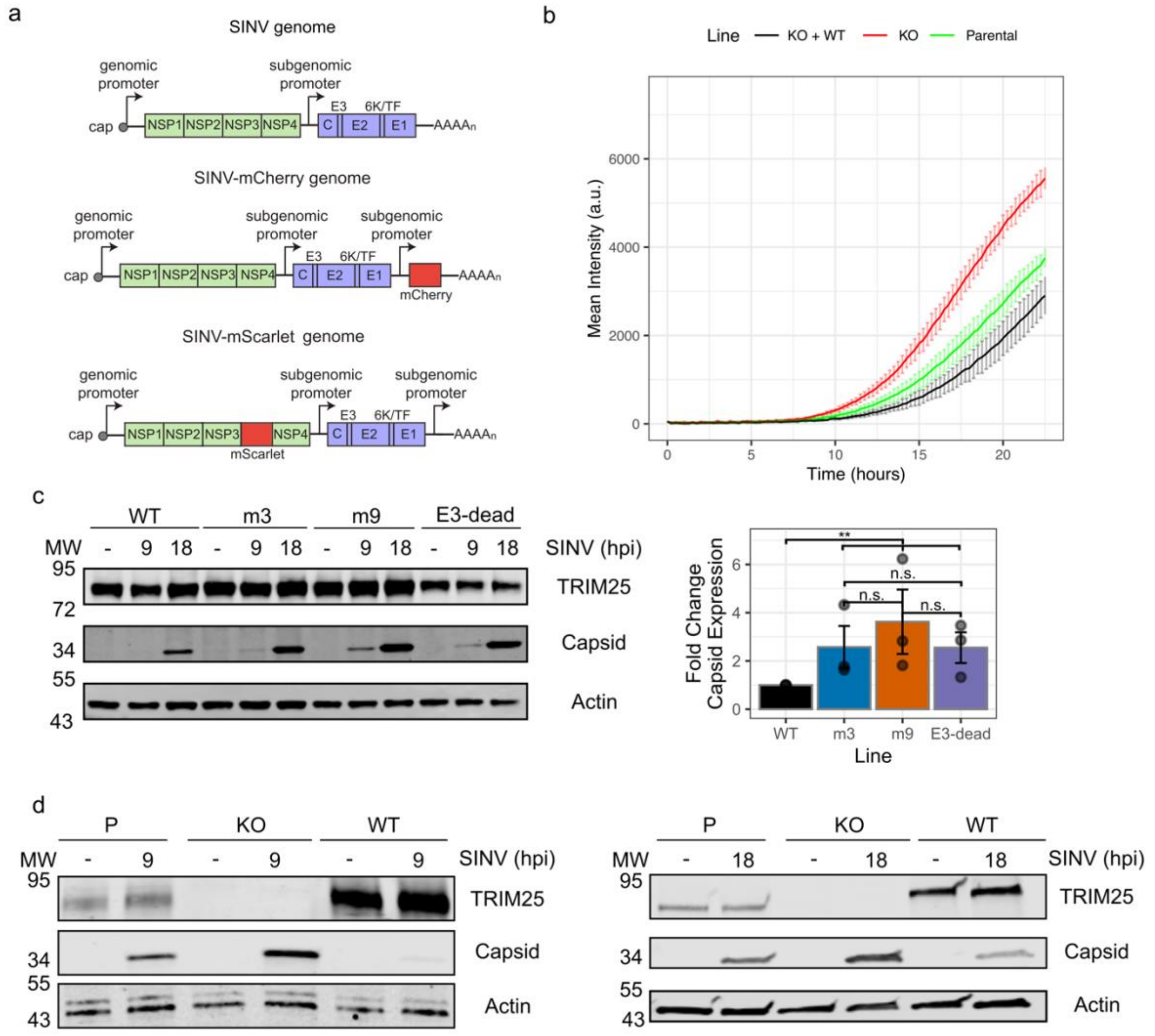

Supplementary Figure 5 (a) Schematic representation of SINV and chimeric SINV-mCherry and SINV-nsp3-scarlet genomes. **(b)** Red fluorescence signal in the parental cell line (P, HEK293. green), the TRIM25 knockout (KO, red), and the cell line rescued using TRIM25-WT (black) infected with SINV-nsp3-scarlet. Fluorescence was measured every 15 min in a plate reader with atmospheric control (5% CO_2 and 37C). The fluorescence is shown as the mean ± SD of six independent infections. **(c)** Western blot analysis of lysates from the different mutant cell lines infected with SINV at different time points post infection (left panel). Quantification of capsid levels of three independent replicates (n=3; right panel). Statistical differences are based on a two-tailed homoscedastic t-test (***p < 0.001; **p < 0.01; *p < 0.05). No adjustments were made for multiple comparisons. **(d)** Western blot analysis of SINV capsid protein accumulation in displaying the parental cell line (P, HEK293), TRIM25 knockout (KO), and the cell line with protein rescue using TRIM25-WT infected with SINV for 9 or 18 h. Uncropped blots in Source Data.

SUPPLEMENTARY REFERENCES

1. Choudhury, N. R. *et al.* RNA-binding activity of TRIM25 is mediated by its PRY/SPRY domain and is required for ubiquitination. *BMC Biol* **15**, 105 (2017).
2. Lorenz, R. *et al.* ViennaRNA Package 2.0. *Algorithms Mol Biol* **6**, 26 (2011).
3. Short, K. M. & Cox, T. C. Subclassification of the RBCC/TRIM superfamily reveals a novel motif necessary for microtubule binding. *J Biol Chem* **281**, 8970–80 (2006).
4. Trewhella, J. *et al.* 2017 publication guidelines for structural modelling of small-angle scattering data from biomolecules in solution: an update. *Acta Crystallogr D Struct Biol* **73**, 710–728 (2017).
